# MAFLD is associated with lower bone mineral density in patients with type 2 diabetes: an exploratory cross-sectional analysis of a potential indirect association with HOMA-IR

**DOI:** 10.3389/fmed.2026.1789018

**Published:** 2026-05-22

**Authors:** Lu Lin, Yanqian Zhuang, Rongkun Xu, Wencheng Yi, Pin Chen, Wenqing Wang

**Affiliations:** 1 900th Hospital of PLA Joint Logistic Support Force, Fuzong Clinical Medical College of Fujian Medical University, Fuzhou, China; 2 The Second Affiliated Hospital of Fujian Medical University, Quanzhou, China

**Keywords:** bone mineral density, insulin resistance, mediation analysis, metabolic dysfunction-associated fatty liver disease, type 2 diabetes mellitus

## Abstract

**Objective:**

To investigate the association between metabolic dysfunction-associated fatty liver disease (MAFLD) and bone mineral density (BMD) in patients with type 2 diabetes mellitus (T2DM), and to explore whether insulin resistance, as indicated by HOMA-IR, statistically accounts for part of this association.

**Methods:**

This retrospective cross-sectional study included 169 patients with T2DM, comprising 108 men with a mean age of 57.43 ± 11.98 years and 61 women with a mean age of 64.02 ± 13.19 years. Participants were divided according to MAFLD status into the MAFLD group (*n* = 86) and the non-MAFLD group (*n* = 83). Demographic, anthropometric, metabolic, and available BMD T-score data were collected. Multivariable linear regression was used to evaluate the associations of MAFLD status and severity with BMD. FIB-4 was assessed as a supplementary non-invasive fibrosis-related index. An exploratory bootstrap-based cross-sectional statistical mediation analysis was performed to estimate the indirect effect of MAFLD on BMD through HOMA-IR.

**Results:**

Compared to the non-MAFLD group, patients in the MAFLD group had significantly lower BMD T-scores (−1.12 ± 1.16 vs. −0.50 ± 1.07, *P* < 0.001). Multivariable regression analysis showed that MAFLD was inversely associated with BMD after adjusting for age, sex and BMI (Model 3: β = −0.562, *P* = 0.003), and the association remained after further including HOMA-IR (Model 4, direct effect: β = −0.545, *P* = 0.006). Bootstrap-based cross-sectional statistical mediation analysis showed a small but statistically significant indirect effect of MAFLD on BMD through HOMA-IR (β = −0.070, 95% CI: −0.142 to −0.015), accounting for 11.4% of the total association. FIB-4 was not independently associated with BMD. In addition, the prevalence of osteopenia/osteoporosis (low bone mass) was higher in the MAFLD group than in the non-MAFLD group (59.0% vs. 33.7%, *P* = 0.002).

**Conclusion:**

In this exploratory single-center cross-sectional study of patients with T2DM, MAFLD was associated with lower BMD. The observed indirect effect via HOMA-IR was modest and should be interpreted as a cross-sectional statistical finding suggestive of a potential pathway; it does not establish temporal sequence, causality, or intervention utility.

## 1 Introduction

Type 2 diabetes mellitus (T2DM) is a chronic metabolic disease with a high global prevalence (1). As well as affecting the cardiovascular, cerebrovascular and renal systems, it is frequently accompanied by abnormalities in bone metabolism, notably a significantly increased risk of osteoporosis and fractures (2). In recent years, as our understanding of metabolic diseases has deepened, it has been suggested that Metabolic Dysfunction-Associated Fatty Liver Disease (MAFLD)—a liver condition closely linked to insulin resistance and metabolic dysregulation—is strongly associated with T2DM and exhibits an extremely high prevalence among T2DM patients (3).

Although the conventional view was that patients with T2DM might have normal or even elevated bone mineral density (BMD) (4), growing evidence indicates that these patients still experience reduced bone quality and an increased risk of fracture, highlighting the particularity of bone metabolism abnormalities in T2DM (5). Meanwhile, MAFLD is recognized not only as a localized hepatic manifestation, but also as an integral component of systemic metabolic disturbance, and its relationship with bone metabolism is becoming an increasingly popular research topic (6, 7).

Existing research suggests that MAFLD may influence bone metabolism through multiple mechanisms, including systemic inflammation, oxidative stress, lipotoxicity (8) and insulin resistance (9). Insulin resistance is a common pathophysiological basis for both MAFLD and T2DM (10) and promotes intrahepatic fat accumulation. It may also directly or indirectly disrupt the balance between osteoblasts and osteoclasts, thereby affecting bone remodeling (11). However, the association between MAFLD and BMD within the T2DM population is unclear, as is the question of whether insulin resistance statistically accounts for part of this association.

However, the relationship between MAFLD and bone mineral density remains controversial, particularly in individuals with T2DM. Previous studies have reported inverse, null, and occasionally even positive associations, which may reflect differences in ethnicity, sex and menopausal status, adiposity, diagnostic criteria, skeletal sites assessed, and the degree of adjustment for confounding factors. Therefore, the present study should be interpreted as an exploratory analysis in a single-center T2DM cohort rather than definitive evidence of a causal relationship.

This study aimed to examine the association between MAFLD and BMD in patients with T2DM and to explore whether insulin resistance statistically accounts for part of this association. Given the cross-sectional design, the analyses were intended to generate hypotheses rather than establish causality.

## 2 Materials and methods

### 2.1 Terminology note

We applied the 2020 diagnostic criteria for MAFLD (hepatic steatosis plus overweight/obesity, type 2 diabetes, or metabolic dysregulation) (12). Since 2023, a multisociety Delphi consensus has recommended steatotic liver disease (SLD) as an overarching term and metabolic dysfunction-associated steatotic liver disease (MASLD) as the replacement term for NAFLD (13). Because participant enrollment and variable collection in the present study (2021–2023) were designed around the MAFLD framework, we report and discuss findings using the term MAFLD. Readers should be aware that MAFLD and MASLD are conceptually similar but not identical constructs; therefore, direct comparisons across studies using different nomenclature/criteria should be made with caution.

### 2.2 Study design and population

We conducted a retrospective observational study. Medical records of patients with type 2 diabetes mellitus who visited or were admitted to the 900th Hospital of the PLA Joint Logistic Support Force between March 2021 and March 2023 were reviewed. After eligibility screening, 169 patients were included and divided according to MAFLD status into the MAFLD group (*n* = 86; mean age, 58.12 ± 12.52 years) and the non-MAFLD group (*n* = 83; mean age, 61.55 ± 12.91 years). Ethics approval was obtained from the Institutional Review Board of the 900th Hospital of the PLA Joint Logistic Support Force Hospital (Approval No. 2025-138). Data extraction and analysis were performed after approval. The requirement for informed consent was waived by the IRB due to the retrospective design, and all data were de-identified before analysis.

In order to clarify the association between MAFLD and BMD, strict exclusion criteria were established to minimize interference from other known factors that affect bone metabolism. These criteria included: (1) other clear etiologies of chronic liver disease, such as positive serological markers for hepatitis B or C, autoimmune liver disease, alcoholic liver disease (alcohol intake >30 g/day) or suspected drug-induced liver injury; (2) severe renal insufficiency, defined as an estimated glomerular filtration rate (eGFR) of < 30 ml/min/1.73 m^2^; (3) patients with a confirmed diagnosis of osteoporosis who were currently receiving anti-osteoporosis medications (e.g., bisphosphonates, teriparatide or denosumab); (4) continuous use of glucocorticoids prior to enrolment or use of other medications known to affect bone metabolism (e.g., antiepileptic drugs or excessive thyroid hormone); (5) pregnant or lactating women; (6) presence of systemic diseases such as malignant tumors, hyperthyroidism or hyperparathyroidism.

### 2.3 Diagnostic criteria and grouping

MAFLD was diagnosed strictly in accordance with the 2020 International Expert Consensus Statement (12). All patients underwent abdominal ultrasonography, which was performed and interpreted independently by two senior ultrasonographers who were blinded to the clinical data. The ultrasonographic diagnostic criteria for hepatic steatosis were diffuse enhancement of liver parenchyma echogenicity (“bright liver”), which was higher than that of the renal cortex and spleen. This was accompanied by posterior echo attenuation and blurring of intrahepatic vessel structures.

Upon confirmation of hepatic steatosis, a diagnosis of MAFLD was made if any one of the following three conditions was met: (1) Overweight/obesity: Body Mass Index (BMI) of at least 23 kg/m^2^; (2) Type 2 diabetes; (3) For individuals of normal weight (BMI of less than 23 kg/m^2^), the presence of at least two additional metabolic abnormalities was required. Waist circumference: Male ≥ 90 cm, female ≥ 80 cm (based on Asian standards); Blood pressure: systolic blood pressure (BP) ≥ 130 mmHg or diastolic BP ≥ 85 mmHg, or ongoing antihypertensive treatment; Plasma triglycerides (TG): ≥ 1.70 mmol/L, or ongoing lipid-lowering treatment; High-density lipoprotein cholesterol (HDL-C): Male < 1.0 mmol/L; Female < 1.3 mmol/L; Fasting plasma glucose: between 5.6 and 6.9 mmol/L (i.e., prediabetes). Based on these criteria, patients were categorized into the MAFLD group (*n* = 86) and the non-MAFLD group (*n* = 83).

The ultrasonographers graded the severity of MAFLD using a standard semi-quantitative method: Grade 0 (none): Normal liver echogenicity. Grade 1 (mild): Mild increase in liver echogenicity; normal visualization of intrahepatic vessel structures; minimal posterior attenuation. Grade 2 (moderate): Moderate increase in liver echogenicity; impaired visualization of intrahepatic vessel structures; mild posterior attenuation. Grade 3 (severe): A marked increase in liver echogenicity, significant posterior echo attenuation and poor or non-visualization of intrahepatic vessel structures. MAFLD severity was independently assessed by two experienced ultrasonographers who were blinded to clinical data, and discrepant assessments were resolved by a third senior ultrasonographer. Because this was a retrospective analysis, the final adjudicated grade rather than the paired independent ratings was retained in the analyzable dataset; therefore, kappa-based inter-rater reliability could not be calculated retrospectively.

### 2.4 Data collection

During the data collection phase, trained research staff collected detailed baseline information from all participants through face-to-face interviews combined with electronic medical record systems. This included demographic data (e.g., age and sex), personal history and a detailed account of current and past medical history. Anthropometric measurements were performed by dedicated personnel using standardized instruments. Height and weight were measured using calibrated stadiometers and scales with participants standing barefoot and wearing light clothing. BMI was then calculated. All participants provided fasting venous blood samples from the antecubital vein the morning after admission, following at least eight hours of fasting. These samples were sent to the hospital's central laboratory for uniform analysis using automated biochemical analysers. Measured parameters included glucose metabolism markers: fasting plasma glucose (FPG), glycated hemoglobin (HbA1c), fasting insulin (FINS) and fasting C-peptide (FCP); lipid profile: total cholesterol (TC), TG, low-density lipoprotein cholesterol (LDL-C) and HDL-C; liver and kidney function markers: Alanine aminotransferase (ALT), aspartate aminotransferase (AST), gamma-glutamyl transferase (GGT), total bilirubin (TBil), total protein (TP), blood urea nitrogen (BUN), serum creatinine (Scr) and uric acid (UA). Additionally, serum calcium levels were measured. The degree of insulin resistance was calculated using the Homeostasis Model Assessment of Insulin Resistance (HOMA-IR) formula: HOMA-IR = [FPG (mmol/L) × FINS (μU/mL)] / 22.5. In addition, the Fibrosis-4 (FIB-4) index was calculated as a non-invasive surrogate marker of liver fibrosis using the following formula: FIB-4 = age (years) × AST (U/L) / [platelet count (10^∧^9/L) × √ALT (U/L)].

BMD was measured using dual-energy X-ray absorptiometry (DXA) with the same Hologic Discovery densitometer by a fixed specialized technician following standard quality-control protocols. Because only one retained BMD T-score variable was available in the retrospective database, this value was used as the primary BMD outcome. Site-specific T-scores for the lumbar spine, femoral neck, and total hip were unavailable; therefore, the outcome should be interpreted as a general retained T-score measure rather than a site-specific estimate.

### 2.5 Statistical analysis

Continuous variables with a normal distribution are presented as the mean ± standard deviation and compared using an independent samples *t*-test. Non-normally distributed variables are presented as the median (interquartile range) and compared using a Mann–Whitney *U*-test. Categorical variables are presented as the frequency (percentage) and compared using a chi-squared test. Hierarchical multivariable linear regression models were used to analyse the association between MAFLD status/severity and BMD T-scores, with stepwise adjustment for confounders (Model 1: unadjusted; Model 2: adjusted for age and sex; Model 3: Model 2 + BMI; Model 4: Model 3 + HOMA-IR). To assess the validity of the regression models, we performed several diagnostic checks. First, we examined multicollinearity among the predictor variables using the variance inflation factor (VIF), with VIF values greater than 5 indicating potential multicollinearity. Second, residuals were assessed for normality and homoscedasticity to ensure that the assumptions of linear regression were met. Sensitivity analyses were conducted to evaluate the robustness of the results: First, we reported the total effect model, excluding HOMA-IR (as it is a statistical mediator) to capture the direct association between MAFLD and BMD. Then, we reported the direct effect model with HOMA-IR included to assess the effect after adjusting for the statistical mediator. Finally, we presented the exploratory statistical mediation analysis separately, with a more cautious explanation of the indirect effect and the proportion statistically accounted for by HOMA-IR. The PROCESS macro with 5,000 bootstrap samples was used for an exploratory cross-sectional statistical mediation analysis to estimate the indirect effect of MAFLD on BMD through HOMA-IR, including the total effect, direct effect, indirect effect, and proportion statistically accounted for by HOMA-IR. Because MAFLD, HOMA-IR, and BMD were measured concurrently, this analysis was interpreted as a statistical decomposition of the observed association rather than as evidence of causal mediation or temporal ordering. All statistical analyses were performed using SPSS software (IBM Corp., Armonk, NY, USA), with a *P* value of less than 0.05 being considered statistically significant. As a supplementary analysis, the association between FIB-4 and BMD T-score was evaluated using linear regression models with sequential adjustment for age, sex, and BMI. For an additional categorical analysis, the available BMD T-score was classified as normal (T-score ≥ −1.0), osteopenia (−2.5 < T-score < −1.0), or osteoporosis (T-score ≤ −2.5). The distribution of these categories, as well as the prevalence of low bone mass (osteopenia/osteoporosis), was compared between the MAFLD and non-MAFLD groups.

Given the modest sample size and retrospective design, the primary multivariable models were kept parsimonious. Several clinically important covariates, including antidiabetic medication use, vitamin D and parathyroid hormone status, menopausal status, physical activity, and smoking, were unavailable and could not be included. Therefore, residual confounding may be substantial, and the regression and mediation results should be interpreted as exploratory.

## 3 Results

### 3.1 Study population overview and baseline characteristics

**Figure 1:**
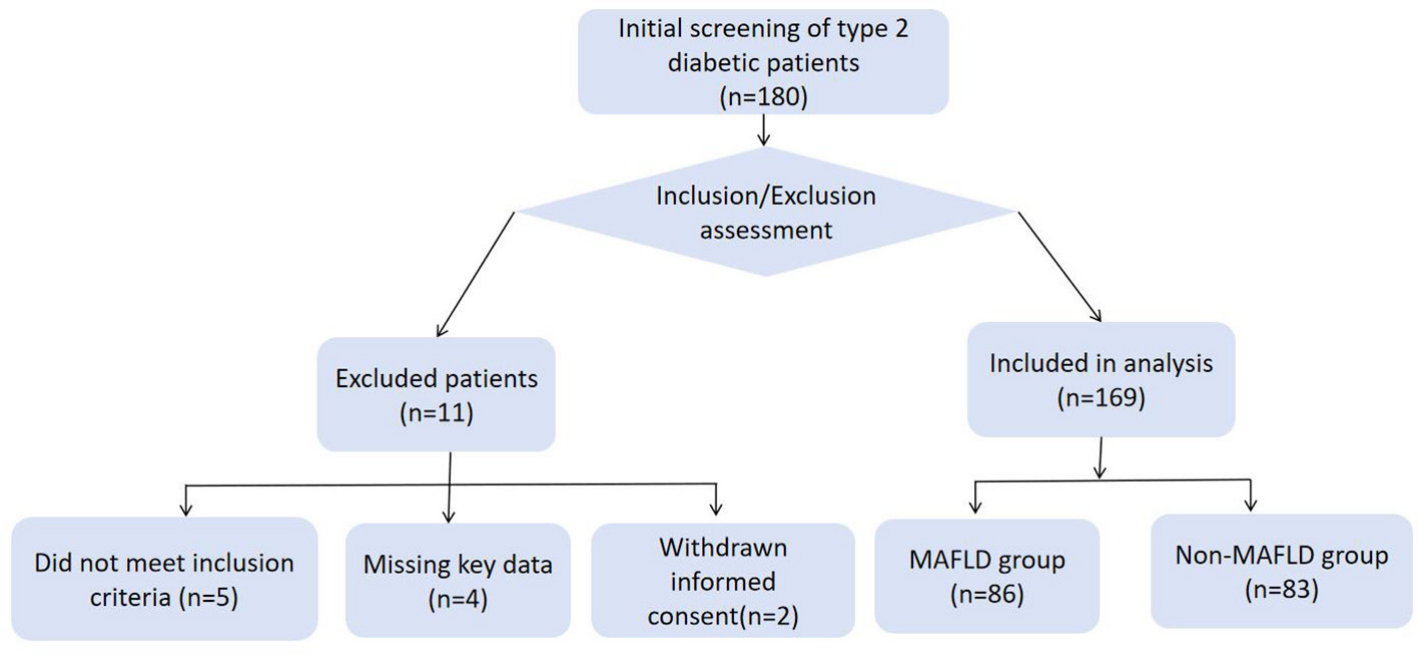
Study population flowchart.

This study included 169 patients with T2DM, who were divided into the MAFLD group (*n* = 86; mean age, 58.12 ± 12.52 years) and the non-MAFLD group (*n* = 83; mean age, 61.55 ± 12.91 years). Age did not differ significantly between the two groups (*P* = 0.081). The participant selection flowchart is shown in Figure 1.

**Figure 2:**
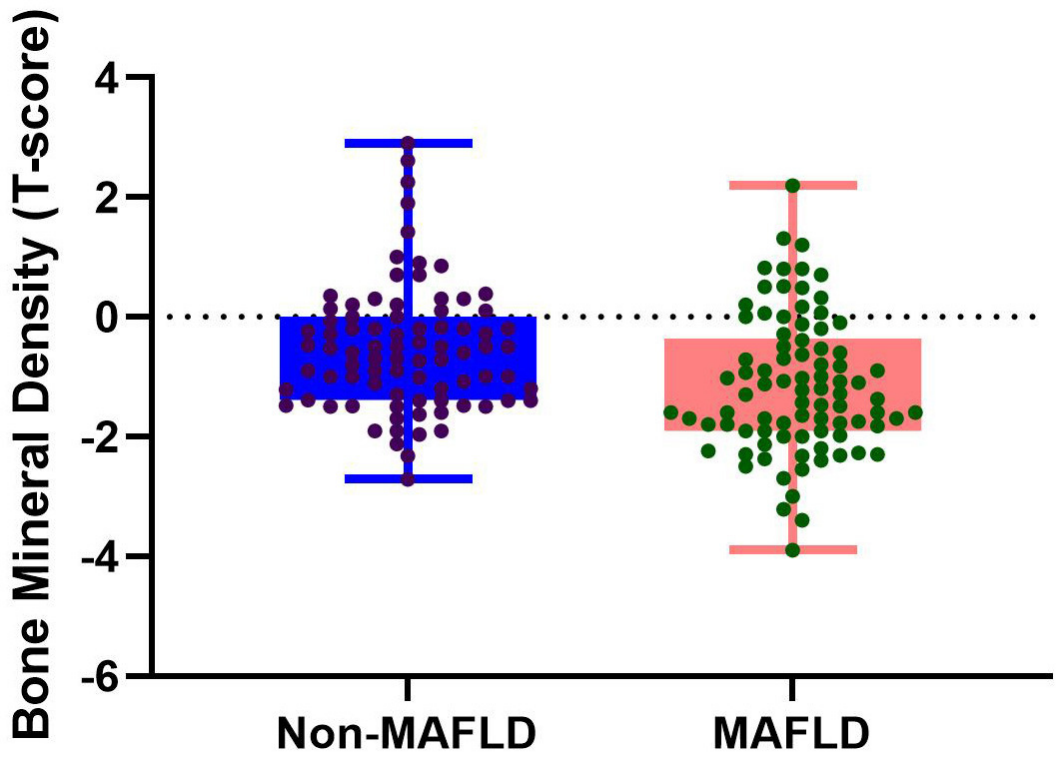
Distribution of bone mineral density T-scores in the MAFLD group vs. the non-MAFLD group (box plot).

A comparison of the baseline data (see Table 1) revealed that patients in the MAFLD group exhibited more severe abnormalities in multiple metabolic indicators. Compared to the non-MAFLD group, patients in this group had significantly higher body weight, BMI, visceral fat area and fat index. Regarding glucose metabolism, the MAFLD group also had significantly higher FPG, FINS, and HOMA-IR levels. Furthermore, the MAFLD group had significantly higher diastolic blood pressure, triglyceride levels, BUN, Scr, urinary albumin-to-creatinine ratio, and liver enzyme levels (ALT, AST, and GGT), while HDL-C and total protein levels were significantly lower. Most critically, the BMD T-score was significantly lower in the MAFLD group than in the non-MAFLD group (−1.12 ± 1.16 vs. −0.50 ± 1.07, *P* < 0.001), as can be seen in the box plot in Figure 2.

**Table 1:** The baseline characteristics of the two groups of participants.

Characteristic	Non-MAFLD group (*N* = 83)	MAFLD group (*N* = 86)	*P* value
Demographics			
Age (years)	61.55 ± 12.91	58.12 ± 12.52	0.081
Male sex, *n* (%)	52 (62.7%)	56 (65.1%)	0.74
Anthropometrics			
Weight (kg)	59.50 (55.25, 60.00)	72.00 (64.50, 78.00)	<0.001
BMI (kg/m^2^)	22.96 (21.05, 25.86)	25.38 (24.11, 29.46)	<0.001
Visceral fat area (cm^2^)	99.60 (59.00, 137.50)	121.00 (83.7, 158.00)	<0.001
Fat index	7.12 ± 2.74	8.42 ± 2.63	0.004
Blood pressure			
SBP (mmHg)	135.25 ± 13.55	137.69 ± 16.04	0.49
DBP (mmHg)	74.25 ± 10.24	83.31 ± 9.37	0.03
Glucose metabolism			
FPG (mmol/L)	8.62 (5.85, 11.48)	9.14 (5.89, 12.11)	0.005
HbA1c (%)	8.70 (7.40, 10.9)	8.95 (8.13, 10.8)	0.17
FINS (μU/mL)	8.29 (7.63, 13.27)	20.11 (15.53, 23.92)	<0.001
FCP (ng/mL)	1.24 ± 0.81	1.58 ± 0.87	0.27
HOMA-IR	4.21 ± 1.25	7.32 ± 2.94	<0.001
Lipid profile			
TC (mmol/L)	5.19 ± 2.12	4.93 ± 1.38	0.60
TG (mmol/L)	1.45 (1.01, 1.99)	2.08 (1.31, 3.02)	<0.001
LDL-C (mmol/L)	2.39 ± 0.54	2.67 ± 1.38	0.16
HDL-C (mmol/L)	1.08 (1.03, 1.47)	0.96 (0.68, 1.26)	0.02
Apolipoprotein A1(g/L)	1.09 ± 0.18	1.03 ± 0.25	0.84
Apolipoprotein B (g/L)	1.05 (0.96, 3.27)	0.92 (0.69, 1.04)	0.43
Renal & liver function			
BUN (mmol/L)	4.90 (4.35, 6.70)	6.60 (4.70, 8.50)	0.002
UA (μmol/L)	305.30 (285.45, 383.20)	298.40 (227.50, 355.85)	0.22
Scr (μmol/L)	74.0 (59.75, 98.18)	87.05 (60.03,148.75)	0.02
UACR (mg/mmol)	1.09 (0.81, 2.04)	39.45 (6.88, 158.45)	0.003
ALT (U/L)	15.4 (11.7, 23.5)	23.0(16.83, 35.30)	<0.001
AST (U/L)	15.60 (13.00, 21.10)	19.00 (15.33, 25.75)	0.002
GGT	16.75 (15.38, 36.35)	22.00 (16.00,53.00)	0.004
Total bilirubin (μmol/L)	7.14 ± 4.20	9.76 ± 4.63	<0.001
Total protein (g/L)	71.00 (63.18, 80.70)	65.90 (64.20, 70.35)	0.007
**FIB-4 index**	1.14 (0.83, 1.54)	1.11 (0.82, 1.56)	0.784
Bone metabolism			
Serum calcium (mmol/L)	2.39 (2.28, 2.45)	2.21 (2.19, 2.34)	0.04
Bone mineral density (T-score)	−0.50 ± 1.07	−1.12 ± 1.16	<0.001
**Bone status**, ***n*** **(%)**			0.002
Normal BMD	55 (66.3%)	34 (41.0%)	
Osteopenia	27 (32.5%)	42 (50.6%)	
Osteoporosis	1 (1.2%)	7 (8.4%)	
**Low bone mass (osteopenia/osteoporosis)**, ***n*** **(%)**	28 (33.7%)	49 (59.0%)	0.002
Comorbidities, *n* (%)			
Hypertension	46 (55.4%)	48 (55.8%)	0.96
Cerebral infarction	19 (22.1%)	20 (24.1%)	0.76
Diabetic retinopathy	28 (33.7%)	19 (22.1%)	0.09
Diabetic peripheral neuropathy	52 (62.7%)	43 (50.0%)	0.10
Coronary heart disease	16 (19.3%)	12 (14.0%)	0.35
Arteriosclerosis	50 (60.2%)	51 (59.3%)	0.90

MAFLD, Metabolic dysfunction-associated fatty liver disease; BMI, Body mass index; SBP, Systolic blood pressure; DBP, Diastolic blood pressure; FPG, Fasting plasma glucose; HbA1c, Glycated hemoglobin; FINS, Fasting insulin; FCP, Fasting C-peptide; HOMA-IR, Homeostatic model assessment of insulin resistance; TC, Total cholesterol; TG, Triglycerides; LDL-C, Low-density lipoprotein cholesterol; HDL-C, High-density lipoprotein cholesterol; Apo, Apolipoprotein; ALT, Alanine aminotransferase; AST, Aspartate aminotransferase; GGT, Gamma-glutamyl transferase; BUN, Blood urea nitrogen; UA, Uric acid; Scr, Serum creatinine; UACR, Urinary albumin-to-creatinine ratio. Data are presented as mean ± standard deviation, median (interquartile range), or number (percentage). *P* values were derived from Student's *t*-test (for normally distributed data), Mann-Whitney *U*-test (for non-normally distributed data), or Chi-squared test (for categorical data). Significant *P* values (< 0.05) are highlighted in bold. Bone status was classified according to the available BMD T-score as normal (T-score ≥ −1.0), osteopenia (−2.5 < T-score < −1.0), or osteoporosis (T-score ≤ −2.5). Percentages for bone status categories were calculated among participants with available BMD T-scores.

In an additional categorical analysis based on the available BMD T-scores, the distribution of normal BMD, osteopenia, and osteoporosis differed significantly between the two groups (*P* = 0.002). Among participants with available T-scores, osteopenia was observed in 42/83 (50.6%) patients in the MAFLD group and 27/83 (32.5%) in the non-MAFLD group, while osteoporosis was observed in 7/83 (8.4%) and 1/83 (1.2%), respectively. When osteopenia and osteoporosis were combined as low bone mass, the prevalence was higher in the MAFLD group than in the non-MAFLD group [49/83 (59.0%) vs. 28/83 (33.7%), *P* = 0.002].

### 3.2 Association analysis between MAFLD and bone mineral density

To clarify the relationship between MAFLD and BMD, we performed multivariable linear regression analyses (Table 2). MAFLD status (yes vs. no) was inversely associated with BMD T-score in the unadjusted model (Model 1: β = −0.615, *P* < 0.001) and remained significant after adjusting for age and sex (Model 2: β = −0.604, *P* = 0.001) and further adjusting for BMI (Model 3, total effect model excluding the statistical mediator: β = −0.562, *P* = 0.003). After additionally including HOMA-IR in the model (Model 4, direct effect: β = −0.545, *P* = 0.006), the association persisted. MAFLD severity (per 1-grade increase) showed an inverse association with BMD in Models 1–3 (Model 3: β = −0.188, *P* = 0.047), but this association was attenuated and became non-significant after adding HOMA-IR (Model 4: β = −0.170, *P* = 0.102), suggesting that insulin resistance may partly explain the link between MAFLD severity and BMD.

**Table 2:** Association between MAFLD status, MAFLD severity and bone mineral density (T-score).

Exposure variable	Model	Adjustment variables	β (95% CI)	SE	Standardized β	*P* value
MAFLD (Yes vs. No)	1	Unadjusted	−0.615 (−0.950, −0.280)	0.170	−0.270	<0.001
	2	+ Age, Sex	−0.604 (−0.942, −0.266)	0.172	−0.265	0.001
	3	+ Age, Sex, BMI	−0.562 (−0.926, −0.198)	0.185	−0.246	0.003
	4	+ Age, Sex, BMI, HOMA-IR	−0.545 (−0.932, −0.158)	0.196	−0.239	0.006
MAFLD Severity (per 1-grade increase)	1	Unadjusted	−0.223 (−0.385, −0.060)	0.082	−0.205	0.007
	2	+ Age, Sex	−0.218 (−0.384, −0.053)	0.084	−0.201	0.010
	3	+ Age, Sex, BMI	−0.188 (−0.373, −0.003)	0.094	−0.173	0.047
	4	+ Age, Sex, BMI, HOMA-IR	−0.170 (−0.375, 0.034)	0.103	−0.157	0.102

MAFLD severity coding: 0 = absent, 1 = mild, 2 = moderate, 3 = severe. The β coefficient denotes: (1) the difference in bone mineral density T-scores between MAFLD and non-MAFLD patients; (2) the change in bone mineral density T-scores per one-level increase in MAFLD severity. CI denotes confidence interval.

As an additional liver-related quantitative parameter, FIB-4 was examined in relation to BMD (Supplementary Table S1). In the overall cohort, FIB-4 was not significantly associated with BMD in the unadjusted model (Model 1: β = 0.277, *P* = 0.053), nor after adjustment for age and sex (Model 2: β = 0.281, *P* = 0.122) or further adjustment for BMI (Model 3: β = 0.320, *P* = 0.077). These findings suggest that, in the present cohort, the observed association between MAFLD and lower BMD was not paralleled by a significant association between FIB-4 and BMD.

Site-specific analyses using lumbar spine, femoral neck, or total hip T-scores could not be performed because these individual DXA site-specific values were not available in the retrospective analyzable dataset.

Because FPG is a component of HOMA-IR, we further evaluated whether additional adjustment for FPG materially changed the direct-effect estimate. In the expanded model including MAFLD, age, sex, BMI, HOMA-IR, and FPG, all VIF values remained below 5, suggesting no serious multicollinearity. The association between MAFLD and lower BMD remained materially unchanged after additional adjustment for FPG (β = −0.527, 95% CI: −0.923 to −0.131, *P* = 0.009), compared with the model adjusted for age, sex, BMI, and HOMA–IR (β = −0.518, 95% CI: −0.909 to −0.128, *P* = 0.010).

### 3.3 Exploratory cross-sectional statistical mediation analysis involving HOMA-IR

To further explore a potential pathway linking MAFLD and lower BMD, we conducted an exploratory cross-sectional statistical mediation analysis (Table 3). HOMA-IR showed a small but statistically significant indirect effect in the association between MAFLD and BMD. The total association between MAFLD and BMD was β = −0.615, of which the estimated indirect effect via HOMA-IR was −0.070, corresponding to 11.4% of the total association. Given the cross-sectional design and simultaneous measurement of MAFLD, HOMA-IR, and BMD, this finding should be interpreted as a statistical indication of a potential pathway rather than confirmation that insulin resistance causally mediates the association between MAFLD and reduced BMD.

**Table 3:** Exploratory cross-sectional statistical mediation analysis of the association between MAFLD status and bone mineral density (T-score) through HOMA-IR.

Pathway	Effect estimate (β)	95% CI	*p* value
Total effect (TE)	−0.615	(−0.950, −0.280)	<0.001
Direct effect (DE)	−0.545	(−0.932, −0.158)	0.006
Indirect effect (IE) via HOMA-IR	−0.070	(−0.142, −0.015)	0.018
Proportion mediated (%)	11.4%	(2.4%, 28.9%)	

The analysis was conducted using a bootstrap approach with 5,000 iterations and adjusted for age, sex, and BMI. The total effect represents the overall association between MAFLD and T-score. The direct effect represents the association after accounting for HOMA-IR. The indirect effect represents the portion of the observed association statistically accounted for by HOMA-IR. Because MAFLD, HOMA-IR, and T-score were measured concurrently, this indirect effect reflects cross-sectional statistical mediation and should not be interpreted as confirming temporal ordering or causality.

## 4 Discussion

This cross-sectional study found that MAFLD was associated with lower BMD T-scores in patients with T2DM. HOMA-IR statistically accounted for a modest proportion of this association. Because MAFLD, HOMA-IR, and BMD were measured simultaneously, the indirect effect should be interpreted as a potential pathway rather than evidence of causal mediation.

In the present study, MAFLD was associated with lower BMD in T2DM patients, which is directionally consistent with some recent reports. For example, Pan et al. (9) found that advanced liver fibrosis in T2DM with MAFLD was linked to higher osteoporosis risk. However, the literature remains inconsistent—previous studies have reported inverse, null, or even positive associations, likely due to differences in ethnicity, body composition, menopausal status, liver disease definitions, BMD sites, and adjustment strategies. Therefore, our findings support a possible negative association in this clinical setting rather than conclusive evidence for all populations (14).

To further address the potential role of liver fibrosis burden, we evaluated FIB-4 as a non-invasive fibrosis-related index. FIB-4 was not independently associated with BMD in the present cohort, suggesting that the observed association between MAFLD and lower BMD may not be primarily explained by fibrosis severity as estimated by FIB-4. Nevertheless, this result should be interpreted cautiously because FIB-4 is an indirect surrogate marker rather than a direct assessment tool (15, 16).

An exploratory finding of this study was that HOMA-IR statistically accounted for 11.4% of the observed association between MAFLD and lower BMD. This cross-sectional indirect effect is compatible with the hypothesis that insulin resistance may be one potential pathway linking MAFLD and skeletal health, but it does not establish that insulin resistance temporally precedes or causally mediates BMD reduction. This hypothesis is biologically plausible, as insulin resistance is a shared pathophysiological feature of MAFLD and T2DM and has been implicated in altered bone remodeling and bone metabolism (17). Zhao et al. (10) emphasized the central role of insulin resistance in metabolic disease and suggested that it may influence bone remodeling directly or indirectly through effects on osteoblast differentiation and function. Karsenty and Khosla further highlighted the close crosstalk between energy metabolism and bone remodeling, proposing that insulin signaling is important for maintaining skeletal homeostasis (11). Additional evidence also supports the broader inflammatory and metabolic context in which insulin resistance may influence diabetic complications. For example, ICAM1-related inflammatory pathways have been associated with diabetes susceptibility, and PTP1B has been implicated in insulin signaling and lipid metabolism in patients with T2DM (18, 19). In addition, studies on glucose-lowering therapies, including SGLT2 inhibitors, highlight the clinical complexity of metabolic treatment in T2DM populations (20). Prospective longitudinal studies with more complete covariate assessment are needed to clarify temporal ordering and underlying mechanisms.

Beyond insulin resistance, other mechanisms are also likely involved, as HOMA-IR statistically accounted for only 11.4% of the association. Chronic low-grade inflammation, oxidative stress, and lipotoxicity accompanying MAFLD may directly or indirectly interfere with bone metabolism (21). In addition, the liver-bone axis has recently attracted attention, with liver-derived factors such as FGF21 and HGF proposed as hepatokines that may regulate bone metabolism remotely (22). These mechanisms may coexist and interact with insulin resistance in relation to bone health (23) From a clinical perspective, our findings support increased awareness of bone health in patients with T2DM and MAFLD. However, the present cross-sectional data do not justify the conclusion that modifying insulin resistance will prevent osteoporosis or improve BMD in this population. Whether interventions targeting insulin resistance, MAFLD, or both can improve skeletal outcomes requires confirmation in prospective longitudinal and interventional studies (24).

This study has several limitations. First, the cross-sectional and single-center design limits causal inference and may introduce selection bias. In addition, participants were recruited from a tertiary hospital in southeastern China, and race/ethnicity was not systematically collected, which may limit generalizability. Second, although we adjusted for age, sex, BMI, and HOMA-IR, the primary models remained relatively parsimonious because of the modest sample size and incomplete availability of clinically important covariates. Some strong determinants of BMD in patients with T2DM were unavailable, including antidiabetic medication classes, vitamin D and parathyroid hormone status, menopausal status, physical activity, and smoking. These factors may influence both bone metabolism and metabolic status/insulin resistance and thus could have confounded the observed association between MAFLD and BMD, as well as the cross-sectional indirect effect involving HOMA-IR. Therefore, the present findings should be interpreted as exploratory associations subject to residual confounding. Third, only a single retained BMD T-score variable was available in the retrospective database, whereas the original site-specific T-scores for lumbar spine L1–L4, femoral neck, and total hip were not retained. Therefore, site-specific sensitivity analyses could not be performed. This is important because lumbar spine and hip BMD may have different determinants and clinical implications. Accordingly, the observed association should not be interpreted as applying equally to all skeletal sites. Finally, MAFLD was diagnosed by ultrasonography rather than by FibroScan, MRI-based techniques, or liver biopsy. Although ultrasound grading was independently performed by two experienced ultrasonographers with adjudication by a third senior ultrasonographer in cases of disagreement, the paired independent grading records were not retained; therefore, inter-rater reliability could not be quantified using kappa statistics.

## 5 Conclusions

In this exploratory single-center cross-sectional study of patients with T2DM, MAFLD was associated with a lower retained BMD T-score. The modest indirect effect through HOMA-IR may suggest a potential pathway, but it does not establish temporal sequence, causality, or intervention utility. Because site-specific DXA T-scores were unavailable, the findings should not be generalized to lumbar spine, femoral neck, or total hip BMD specifically. Larger multicentre longitudinal studies with complete site-specific DXA assessment are needed.

## Data Availability

The original contributions presented in the study are included in the article/Supplementary material, further inquiries can be directed to the corresponding authors.
