# Correction: MAFLD is associated with lower bone mineral density in patients with type 2 diabetes: an exploratory cross-sectional analysis of a potential indirect association with HOMA-IR

**DOI:** 10.3389/fmed.2026.1937985

**Published:** 2026-07-24

**Authors:** Lu Lin, Yanqian Zhuang, Rongkun Xu, Wencheng Yi, Pin Chen, Wenqing Wang

**Affiliations:** 1 900th Hospital of PLA Joint Logistic Support Force, Fuzong Clinical Medical College of Fujian Medical University, Fuzhou, China; 2 The Second Affiliated Hospital of Fujian Medical University, Quanzhou, China

**Keywords:** bone mineral density, insulin resistance, mediation analysis, metabolic dysfunction-associated fatty liver disease, type 2 diabetes mellitus

The funder “Joint Logistic Quality Discipline Fund (Grant Number LQYZ-NFM)” was erroneously included.

The original version of this article has been updated.

