# Supplementary material for: MAFLD is associated with lower bone mineral density in patients with type 2 diabetes: an exploratory cross-sectional analysis of a potential indirect association with HOMA-IR
Source: Front Med (Lausanne). 2026 May 22;13:1789018. doi: 10.3389/fmed.2026.1789018 (PMC13237680; doi:10.3389/fmed.2026.1789018)
Supplement: Supplementary file 1 [file Table_1.doc]

**Supplementary Table S1. Association between FIB-4 and bone mineral density (T-score)**

| **Exposure Variable** | **Model** | **Adjustment Variables** | **β (95% CI)** | **SE** | **Standardized β** | ***P* value** |
| --- | --- | --- | --- | --- | --- | --- |
| FIB-4 (per 1-unit increase) | 1 | Unadjusted | 0.277 (-0.004, 0.558) | 0.142 | 0.152 | 0.053 |
|  | 2 | + Age, Sex | 0.281 (-0.076, 0.638) | 0.181 | 0.154 | 0.122 |
|  | 3 | + Age, Sex, BMI | 0.320 (-0.035, 0.674) | 0.180 | 0.175 | 0.077 |

**Note:** The β coefficient denotes the change in bone mineral density T-score per 1-unit increase in FIB-4. CI denotes confidence interval. Because age is a component of the FIB-4 formula, the age-adjusted models should be interpreted cautiously.
