# Supplementary material for: MAFLD is associated with lower bone mineral density in patients with type 2 diabetes: an exploratory cross-sectional analysis of a potential indirect association with HOMA-IR
Source: Front Med (Lausanne). 2026 May 22;13:1789018. doi: 10.3389/fmed.2026.1789018 (PMC13237680; doi:10.3389/fmed.2026.1789018)
Supplement: Supplementary file 2 [file Table_2.doc]

**Supplementary Table S2. Sensitivity analysis additionally adjusting for FPG**

| **Model** | **Adjustment variables** | **β for MAFLD** | **95% CI** | **P value** | **Maximum VIF** |
| --- | --- | --- | --- | --- | --- |
| Main direct-effect model | Age, sex, BMI, HOMA-IR | -0.518 | -0.909 to -0.128 | 0.010 | 1.30 |
| FPG-adjusted sensitivity model | Age, sex, BMI, HOMA-IR, FPG | -0.527 | -0.923 to -0.131 | 0.009 | 2.07 |

**Note:** FPG was additionally included because it is a component of HOMA-IR. VIF, variance inflation factor. All VIF values were below 5, indicating no serious multicollinearity.
